# Haemoglobin level at birth is associated with short term outcomes and mortality in preterm infants

**DOI:** 10.1186/s12916-014-0247-6

**Published:** 2015-01-27

**Authors:** Jayanta Banerjee, Felix K Asamoah, Devpriya Singhvi, Angela WG Kwan, Joan K Morris, Narendra Aladangady

**Affiliations:** Neonatal Unit, Homerton University Hospital NHS Foundation Trust, Homerton, London, E9 6SR UK; Centre for Paediatrics, Barts and the London School of Medicine and Dentistry, Queen Mary University of London, Mile End Road, London, E1 4NS UK; Portland Hospital, Great Portland Street, London, W1W 5AH UK; Wolfson Institute, Barts and the London School of Medicine and Dentistry, Queen Mary University of London, Charterhouse Square, London, EC1M 6BQ UK; Department of Paediatrics, SDM Medical College and Hospitals, Dharwad, Karnataka India

**Keywords:** Haemoglobin, Mortality, Outcome, Preterm

## Abstract

**Background:**

Blood volume and haemoglobin (Hb) levels are increased by delayed umbilical cord clamping, which has been reported to improve clinical outcomes of preterm infants. The objective was to determine whether Hb level at birth was associated with short term outcomes in preterm infants born at ≤32 weeks gestation.

**Methods:**

Data were collected retrospectively from electronic records: Standardised Electronic Neonatal Database, Electronic Patient Record, Pathology (WinPath), and Blood Bank Electronic Database. The study was conducted in a tertiary perinatal centre with around 5,500 deliveries and a neonatal unit admission of 750 infants per year. All inborn preterm infants of 23 to 32 weeks gestational age (GA) admitted to the neonatal unit from January 2006 to September 2012 were included.

The primary outcomes were intra-ventricular haemorrhage, necrotising entero-colitis, broncho-pulmonary dysplasia, retinopathy of prematurity, and death before discharge. The secondary outcomes were receiving blood transfusion and length of intensive care and neonatal unit days. The association between Hb level (g/dL) at birth and outcomes was analysed by multiple logistic regression adjusting for GA and birth weight (BWt).

**Results:**

Overall, 920 infants were eligible; 28 were excluded because of missing data and 2 for lethal congenital malformation. The mean (SD) GA was 28.3 (2.7) weeks, BWt was 1,140 (414) g, and Hb level at birth was 15.8 (2.6) g/dL.

Hb level at birth was significantly associated with all primary outcomes studied (*P* <0.001) in univariate analyses. Once GA and BWt were adjusted for, only death before discharge remained statistically significant; the OR of death for infants with Hb level at birth <12 g/dL compared with those with Hb level at birth of ≥18 g/dL was 4.1 (95% CI, 1.4–11.6). Hb level at birth was also significantly associated with blood transfusion received (*P* <0.01) but not with duration of intensive care or neonatal unit days.

**Conclusions:**

Low Hb level at birth was significantly associated with mortality and receiving blood transfusion in preterm infants born at ≤32 weeks gestation. Further studies are needed to determine the association between Hb level at birth and long-term neurodevelopmental outcomes.

**Electronic supplementary material:**

The online version of this article (doi:10.1186/s12916-014-0247-6) contains supplementary material, which is available to authorized users.

## Background

Short-term outcomes of a preterm infant are influenced by the gestational age (GA), birth weight (BWt), sex, antenatal factors, and condition of the infant at birth and during the first few hours of life [1]. Interventions in the antenatal period, delivery, and at birth also influence the short-term outcomes of a preterm infant. Chorioamnionitis is associated with intra-ventricular haemorrhage and white matter injury as well as poor neurodevelopmental outcome at 2 years of age [2]. Administration of antenatal steroids in threatened preterm labour significantly improves short-term outcomes [3]. Costeloe et al. [1] reported improved overall survival of extreme preterm infants (23 to 25 weeks gestation) born by caesarean section. Administration of surfactant significantly reduces the severity of respiratory distress syndrome and ventilatory requirement, and improves the overall survival of preterm infants [4,5]. Further, infant low body temperature on admission to the neonatal unit and severe metabolic acidosis during the first 12 hours of age are associated with poor short-term outcomes [6]. Other factors, such as low haematocrit (Hct), mean blood pressure, urine output, and high oxygen requirement during the first 24 hours of age, also influence short-term outcomes of preterm infants [7].

Hct and haemoglobin (Hb) level could be improved by delaying clamping and/or by milking of the umbilical cord in term [8] and preterm [9] infants. In a randomised controlled trial of 46 preterm infants born between 24 and 32 weeks gestation, measured circulating blood volume was higher in infants delivered by delayed cord clamping (≥30 seconds) compared to early cord clamping; the benefit was seen in both vaginal and caesarean section deliveries [10]. Though the increased blood volume is noted soon after delivery, the raised Hb and Hct become apparent after several minutes to hours following birth. Providing additional placental blood to the preterm infant by delaying cord clamping for 30 to 120 seconds appears to be associated with better circulatory stability, less respiratory distress syndrome, less need for blood transfusion, and a lower risk of intra-ventricular haemorrhage (IVH) and necrotising enterocolitis (NEC) [9].

Currently, there is a paucity of evidence regarding the relationship between Hb level at birth and morbidity and mortality of preterm infants irrespective of the mode of delivery and time of umbilical cord clamping. The objectives of our study were to evaluate the relationship between Hb level at birth and primary outcomes of IVH, NEC, broncho-pulmonary dysplasia (BPD), retinopathy of prematurity (ROP), and death before discharge as well as secondary outcomes of receiving red blood cell transfusions, length of intensive care stay, and total neonatal unit days in preterm infants born at ≤32 weeks gestation.

## Methods

The study was conducted in a tertiary referral obstetric and neonatal unit in London, UK, with an average neonatal unit admission of 750 infants per year. All inborn preterm infants of 23 to 32 weeks GA admitted to the neonatal unit between January 2006 and September 2012 were included. Infants with a life-limiting major congenital abnormality (e.g., Trisomy 13 and Trisomy 18) were excluded. Data were collected retrospectively from electronic records: Standardised Electronic Neonatal Database, Electronic Patient Record, Pathology (WinPath), and Blood Bank Electronic Database. Two researchers collected data independently (AK and DPS) and the data were verified by two senior researchers (JB and NA). Patient demographics (GA, BWt, ethnicity, and sex), antenatal details (ante-partum haemorrhage, chorioamnionitis, pre-eclampsia, and foetal distress), mode of delivery (vaginal or caesarean section), Hb level (g/dL) at birth, admission temperature, blood transfusion details, and short-term outcomes (IVH, NEC, ROP, BPD, intensive care and total neonatal unit days, and death before discharge from the neonatal unit) were collected.

Ante-partum haemorrhage was defined as any per-vaginal bleed in the antenatal period, and chorioamnionitis was defined as presence of maternal fever (>38°C), maternal tachycardia (>100 bpm), foetal tachycardia (>160 bpm), foul smelling vaginal discharge, and preterm prolonged (>18 hours) rupture of membranes [11]. Maternal raised blood pressure with proteinuria was recorded as pre-eclampsia and abnormal cardiotocography, and absence or reduced foetal movements warranting urgent obstetric response were recorded as foetal distress. Plastic bags were routinely used along with radiant warmer during stabilisation in the delivery room for babies born at <29 weeks gestation during the study period. Admission temperature was the first recorded temperature on admission to the neonatal unit within the first hour of birth. Hb level at birth was measured from venous blood samples taken while siting an intravenous cannula within the first hour of age using flow cytometry (Beckman Coulter Inc., USA). Level of care was recorded according to the British Association of Perinatal Medicine classification of levels of care [12]. Cranial ultrasound scans were performed by two trained dedicated neonatal sonographers. The final worst result agreed by the attending neonatologist was collected as IVH (Grade 1–4 Papille) [13]. ROP was recorded as present or absent, and according to the International Classification stages 1 to 4 [14]. BPD was defined as oxygen requirement at 36 weeks gestation as defined by the National Institute of Child Health and Human Development [15].

Informed parent consent was not required as this was a retrospective study using anonymised routinely collected patient data. As required by the UK research ethics principles, the study protocol was reviewed by the hospital research and development committee and permission was granted to conduct the study (R&D Reference no. PA 1207). Data was analysed using statistical software STATA 12.0 (STATA Corp LP, Texas, USA). For continuous and for categorical variables *t*-tests and χ^2^ tests were performed, respectively. Hb level at birth was categorised into five groups (<12, 12 to <14, 14 to <16, 16 to <18, and ≥18 g/dL). The association between the Hb level at birth and primary outcomes as well as blood transfusion received was analysed by multiple logistic regression. For infants who survived to discharge, the association between Hb level at birth and length of intensive care and neonatal unit days was analysed by linear regression. As BWt is highly correlated with GA, standardised BWt z-scores were calculated using the 2010 UK-WHO growth chart [16]. GA, standardised BWt, sex, ethnicity, mode of delivery, foetal distress, antepartum haemorrhage, chorioamnionitis, and pre-eclamptic toxaemia were included in the logistic regression models to determine if they were independently associated with the outcomes. Only standardised BWt and GA were significantly associated with the outcomes and were included in all the multivariate models. A *P* value of <0.05 was considered statistically significant.

## Results

**Figure 1:**
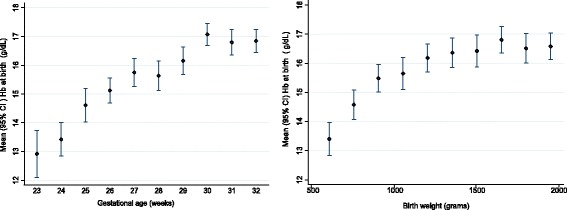
**Haemoglobin at birth in relation to gestational age and birth weight (n = 890).**

Overall, 920 infants were eligible for the study; 28 infants were excluded because of missing Hb level at birth (n = 13) and outcome data (n = 15). Two infants were excluded because of major congenital abnormalities: one Trisomy 13 and one Trisomy 18. In total, 890 infants were included in the analysis with a mean (standard deviation; SD) GA of 28.3 (2.7) weeks and BWt of 1,140 (414) g. The mean (SD) admission temperature was 36.6 (0.7)°C. The mean (SD) Hb level at birth was 15.8 (2.6) g/dL and Hct 46.5 (7.6)%. The Hb level at birth was positively associated with GA up until 30 weeks of gestation and BWt up until 1,500 g (*P* <0.01; Figure 1). The relationship between Hb level at birth and other infant as well as maternal characteristics are presented in Table 1.

**Table 1:** **Association of haemoglobin at birth and maternal and infant characteristics (n = 890)**

**Characteristics (number)**		**Mean (95% CI) Hb at birth (g/dL)**	***P*** **value**
**Sex**	Male (450)	15.9 (15.7–16.2)	0.129
	Female (440)	15.7 (15.4–15.9)	
**Ethnicity**	Caucasian (319)	16.3 (15.9–16.7)	<0.01
	Black (331)	15.2 (14.9–15.5)	
	Asian (129)	15.6 (15.1–16.1)	
	Mixed (111)	16.3 (15.9–16.8)	
**Type of delivery**	Vaginal (362)	15.3 (15.0–15.6)	<0.01
	Caesarean (527)	16.2 (15.9–16.4)	
**Foetal distress**	Yes (107)	14.6 (14.1–15.1)	<0.01
	No (777)	16.0 (15.8–16.1)	
**Antepartum haemorrhage**	Yes (234)	15.2 (14.9–15.6)	<0.01
	No (656)	16.0 (15.8–16.2)	
**Chorioamnionitis**	Yes (252)	15.2 (14.9–15.6)	<0.01
	No (638)	16.0 (15.8–16.2)	
**Pre-eclamptic toxemia**	Yes (261)	16.0 (15.6–16.3)	<0.01
	No (624)	15.7 (15.5–15.9)	

**Figure 2:**
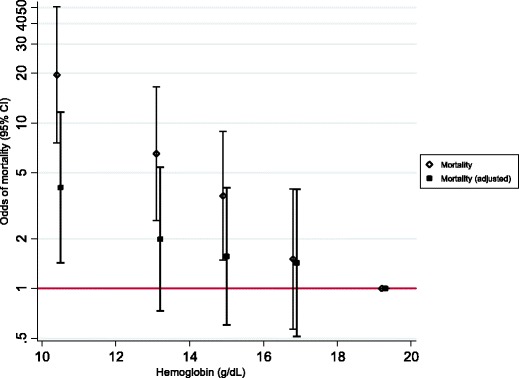
**Association of haemoglobin at birth and death before discharge (n = 890).**

The primary outcomes of IVH, NEC, BPD, ROP, and death before discharge were significantly associated with Hb level at birth (Table 2). Infants with Hb level at birth <12 g/dL compared to those with ≥18 g/dL were at a significant risk of death before discharge (Odds ratio (OR), 19.5; 95% CI, 7.6–50.4; *P* <0.01). After adjusting for GA and BWt, this association reduced but remained statistically significant (OR, 4.1; 95% CI, 1.4–11.6; *P* = 0.01; Table 2 and Figure 2).

**Table 2:** **Relationship between haemoglobin at birth and primary outcomes (n = 890)**

**Short term outcomes**	**Number (%)**	**Haemoglobin at birth (g/dL)**						***P*** **values for trend**
		**OR**	**<12**	**12 to <14**	**14 to <16**	**16 to <18**	**18+**	
		**(95% CI)**	**n = 67**	**n = 124**	**n = 122**	**n = 408**	**n = 169**	
**Deaths before discharge**	103 (11.6)	Crude	19.5 (7.6–50.4)	6.5 (2.6–16.5)	2.8 (1.2–6.7)	1.7 (0.5–5.0)	1	<0.01
		Adjusted^†^	4.1 (1.4–11.6)	2.0 (0.7–5.4)	1.5 (0.6–3.7)	1.7 (0.5–5.7)	1	0.01
**Intraventricular haemorrhage (All grades)**	233 (26.2)	Crude	4.2 (2.2–8.0)	3.6 (2.1–6.2)	2.1 (1.3–3.3)	1.0 (0.5–1.8)	1	<0.01
		Adjusted^†^	1.6 (0.8–3.3)	1.9 (1.0–3.4)	1.5 (1.0–2.4)	0.9 (0.5–1.8)	1	0.53
**Necrotising enterocolitis (All grades)**	195 (21.9)	Crude	2.6 (1.3–5.1)	3.2 (1.8–5.7)	1.7 (1.0–2.8)	1.1 (0.6–2.1)	1	<0.01
		Adjusted^†^	1.0 (0.5–2.2)	1.8 (1.0–3.4)	1.3 (0.8–2.2)	1.2 (0.6–2.3)	1	0.31
**Bronchopulmonary dysplasia**	242 (27.2)	Crude	3.1 (1.7–5.9)	3.1 (1.8–5.3)	2.0 (1.3–3.1)	0.6 (0.3–1.1)	1	<0.01
		Adjusted^†^	0.5 (0.3–1.1)	1.0 (0.5–1.9)	1.2 (0.7–2.0)	0.5 (0.2–1.1)	1	0.64
**Retinopathy of prematurity (All grades)**	185 (20.8)	Crude	3.2 (1.5–6.4)	4.9 (2.7–8.8)	1.9 (1.2–3.2)	0.9 (0.4–1.9)	1	<0.01
		Adjusted^†^	0.7 (0.3–1.5)	1.9 (1.0–3.8)	1.2 (0.7–2.1)	0.9 (0.4–2.0)	1	0.72

^†^Adjusted odds ratio (OR) for gestational age and birth weight.

Hb level at birth was significantly associated with the secondary outcomes of receiving a blood transfusion and duration of intensive care and neonatal unit stay (Table 3). Overall, 518 (58.2%) infants received blood transfusions; the odds of receiving a blood transfusion increased with decreasing Hb level at birth (*P* <0.01) even after adjusting for GA and BWt. For the 787 infants who survived to discharge, the median (IQR) duration of intensive care and total neonatal unit days were 5 (2 to 22) and 42 (27 to 66) days, respectively. Hb level at birth was inversely associated with both intensive care days (*P* <0.01) and total neonatal unit days (*P* = 0.01; Table 3). However, when adjusted for GA and BWt, the association between Hb level at birth and duration of intensive care (*P* = 0.11) as well as total neonatal unit (*P* = 0.17) days were not significant.

**Table 3:** **Relationship between haemoglobin at birth and secondary outcomes (n = 890)**

**Health interventions**	**Number (%)**	**Haemoglobin at birth (g/dL)**						***P*** **values for trend**
			**<12**	**12 to <14**	**14 to <16**	**16 to <18**	**18+**	
			**n = 67**	**n = 124**	**n = 122**	**n = 408**	**n = 169**	
Transfusion* (n = 890)	518 (58.2)	Crude (95% CI)	27.2 (9.4–78.3)	6.8 (4.0–11.7)	5.0 (3.0–8.3)	1.7 (1.2–2.5)	1	<0.01
		Adjusted^†^ (95% CI)	16.6 (3.9–70.5)	3.5 (1.7–7.4)	2.7 (1.4–5.2)	1.6 (1.0–2.5)	1	<0.01
**Infants survived to discharge (n = 787)**								
			**n = 39**	**n = 100**	**n = 106**	**n = 379**	**n = 163**	
**Median length of intensive care days (95% CI)**	787 (88.4)	Median (95% CI)	20 (4.3–35.7)	14.5 (4.3–24.7)	8 (4.6–11.4)	4 ( 3.0–5.0)	4 (3.0–5.0)	<0.01
**Median length of neonatal unit days (95% CI)**	787 (88.4)	Median (95% CI)	44 (29.3–58.7)	53 (43.2–62.8)	48 (43.2–52.8)	37 (341–39.9)	37 (33.6–40.7)	0.01

*Odds ratio, ^†^Adjusted for GA and BWt.

## Discussion

We have shown that lower Hb level at birth was significantly associated with the primary outcome of death before discharge in preterm infants born at ≤32 weeks of gestation independent of GA and BWt. This result is consistent with that observed by Hosono et al. [17], who demonstrated a reduced risk of mortality in infants with Hb level at birth ≥15 g/dL compared to <15 g/dL in a smaller cohort of 54 infants. However, during the development and validation of Clinical Risk Index for Babies score (n = 812 infants born at 23 to 31 weeks of gestation), anaemia in the first 12 hours of life was not found to be significantly associated with mortality in a univariate analysis, and hence was excluded from the final regression analysis [18]. A meta-analysis of studies comparing delayed versus early umbilical cord clamping showed an increased haematocrit mean difference of 3.26% (95% CI, 1.79–4.74) at birth or 1 hour, 5.4% (95% CI, 3.62–7.17) at 4 hours, and 3.28% (95% CI, 1.34–5.22) at 24 hours in the delayed cord clamping group. There was no clear difference in the risk of death (OR, 0.63; 95% CI, 0.31–1.28) between these two groups [9]. The Hb level at birth of infants in our study is comparable to reported Hb levels at birth for preterm infants [19]. The survival of infants in this study (88.4%) is comparable to the Models of OrganiSing Access to Intensive Care (MOSAIC) birth cohort (preterm infants 22 to 31 weeks and 6 days of GA) of 10 geographic European regions (89.5%) [20].

We have shown that lower Hb level at birth was significantly associated with IVH but this was not significant when GA and BWt were adjusted for. The incidence of IVH (26.2%) was comparable to that reported in previous studies [21,22]. Similar to our findings, Hosono et al. [17] reported an increased incidence of IVH in extremely low BWt infants with Hb level at birth <15 g/dL. Other researchers have also demonstrated that low initial haematocrit was associated with higher incidence of IVH [22]. In addition, increased Hb level at birth by delaying umbilical cord clamping has been demonstrated to reduce the risk of IVH (relative risk, 0.59; 95% CI, 0.41–0.85) [9].

Lower Hb level at birth was associated with risk of NEC, ROP, and BPD, although this association was not significant when adjusted for GA and BWt in the present study. Increased Hb level at birth by delaying umbilical cord clamping has been demonstrated to reduce the risk of NEC but not ROP or BPD [9]. The incidences of BPD (all grades), ROP, and NEC in our study are comparable to those in previous studies [23-25]. We speculate that higher Hb level at birth possibly resulted in better haemodynamic stability and reduced severity of cardio-respiratory illness, which in turn reduced the severity of short-term complications of prematurity and improved overall survival in this study.

Overall, 58.2% of infants in our study received a blood transfusion during their hospital stay, which is comparable that in other studies [26]. Rabe et al. [9] reported lower Hb in infants delivered by early cord clamping, and this was associated with an increased need for blood transfusion similar to the present study. There was a negative association with Hb level at birth and duration of intensive care and total neonatal unit admission days, but this association was not significant when adjusted for GA and BWt in this study. Similar to the present study, Hosono et al. [17] reported that the duration of ventilation was higher in extremely low BWt infants with low Hb (<15 g/dL) at birth, but was not significant. Preterm infants with higher Hb level at birth (delivered by delayed umbilical cord clamping) were reported to have reduced requirements of respiratory support and intensive care [9], which is in keeping with the findings of our study. The median total neonatal unit stay (44 days) for infants born with Hb level at birth <12 g/dL was shorter compared to infants born with Hb ≥12 g/dL in this study. This is because of the higher proportion of death in this group of infants; 42% of infants with Hb level at birth <12 g/dL died compared to 9% of infants with Hb level at birth ≥12 g/dL.

One of the limitations of this study was that the actual timing of umbilical cord clamping was not available. However, during the study period, the standard practice was early cord clamping. We also did not collect details of antenatal steroids received by mothers in this study, which is likely to have an impact on the short-term outcomes and mortality of infants studied [3]; however, in our hospital, around 90% of mothers of infants born at ≤32 weeks gestation receive antenatal steroids. The hospital policy regarding neonatal discharge remained same during the study period. However, change in length of stay over the study period was not studied. Further, 28 infants were excluded from the study because of missing data but this constitutes a very small proportion (3%) of the study population, and this should not have significant impact on the study findings. In the present study more infants were born by caesarean section (59.3%), but this is comparable to other specialist perinatal referral centres [1,27].

## Conclusions

To our knowledge, this is the first study reporting a low Hb level at birth as an independent risk factor for mortality and probability of receiving blood transfusion in preterm infants born at ≤32 weeks gestation, irrespective of mode of delivery and time of umbilical cord clamping. Hence, more thought should be given to delayed cord clamping in preterm infants to improve the Hb level at birth, which in turn could reduce mortality and short-term complications of prematurity. The neonatal mortality prediction scores need to be investigated by incorporating Hb level at birth to see whether this increases the robustness of prediction of short-term outcomes and mortality in preterm infants. Further studies are required to investigate the association between Hb level at birth and long-term neurodevelopmental outcomes.

## Abbreviations

BWt: Birth weight
ELBW: Extremely low birth weight
GA: Gestational age
Hb: Haemoglobin
Hct: Haematocrit
IVH: Intraventricular haemorrhage
NEC: Necrotising enterocolitis
OR: Odds ratio
ROP: Retinopathy of prematurity
SD: Standard deviation
